# Near-Infrared Fluorescence Imaging and Photodynamic Therapy for Liver Tumors

**DOI:** 10.3389/fonc.2021.638327

**Published:** 2021-02-25

**Authors:** Masaki Kaibori, Hisashi Kosaka, Kosuke Matsui, Morihiko Ishizaki, Hideyuki Matsushima, Takumi Tsuda, Hidehiko Hishikawa, Tadayoshi Okumura, Mitsugu Sekimoto

**Affiliations:** Department of Surgery, Kansai Medical University, Osaka, Japan

**Keywords:** near-infrared fluorescence imaging, liver surgery navigation, photodynamic therapy, liver tumors, indocyanine green lactosome

## Abstract

Surgery with fluorescence equipment has improved to treat the malignant viscera, including hepatobiliary and pancreatic neoplasms. In both open and minimally invasive surgeries, optical imaging using near-infrared (NIR) fluorescence is used to assess anatomy and function in real time. Here, we review a variety of publications related to clinical applications of NIR fluorescence imaging in liver surgery. We have developed a novel nanoparticle (indocyanine green lactosome) that is biocompatible and can be used for imaging cancer tissues and also as a drug delivery system. To date, stable particles are formed in blood and have an ~10–20 h half-life. Particles labeled with a NIR fluorescent agent have been applied to cancer tissues by the enhanced permeability and retention effect in animals. Furthermore, this article reviews recent developments in photodynamic therapy with NIR fluorescence imaging, which may contribute and accelerate the innovative treatments for liver tumors.

## Introduction

Many imaging tools, including ultrasonography (US), positron emission tomography, computed tomography (CT), and magnetic resonance imaging (MRI), are used and necessary for recent hepatic surgery. However, these imaging treatments can be insufficient for detecting smaller tumors, which require more high spatial resolution than these methods have. For clinical use, real-time cancer detection with fluorescence probes, such as indocyanine green (ICG) and the porphyrin precursor, 5-aminolevulinic acid (5-ALA), is an important topic. ICG and 5-ALA are appropriated as fluorescence imaging (FI) agents, but their application is inadequate for certain diseases. After ICG injection, intraoperative fluorescent angiography has been performed to evaluate the patency of coronary artery bypass grafts (1–4). The use of intraoperative FI can reportedly allow better visualization during hepatobiliary surgery (5). ICG can bind to plasma proteins and affect its fluorescence, emitting near-infrared (NIF) light (6, 7). Proteins in human bile also bind to ICG (8). Prior to hepatic surgery, ICG is administered to measure the ICG retention time (ICGR15; at 15 min), estimating the maximum hepatic volume that can be safely resected (9, 10). Many high-volume hepatectomy centers in Japan are beginning to use ICG FI for intraoperative navigation, such as visualizing small liver tumors, cholangiography, and imaging hepatic segments.

Remarkable advances in photodynamic therapy (PDT) for liver tumors have also been reported. Development of molecular probes based on nanoparticles, polymer micelles that use the characteristics of neovascularization, is currently underway. By including various signaling agents (e.g., radioisotopes, fluorescent agents, magnetic substances) in the nanoparticles, diagnosis can be made by molecular imaging of new vascular tissue. If a therapeutic agent is included, this method may also provide a drug delivery system. Theranostics (a fusion of “diagnostics” and “therapy”) can be realized (11) and may lead to ultra-early cancer diagnosis and treatment.

We used the enhanced permeability and retention characteristics of neovascularization (12), which are prominent in inflammatory diseases such as rheumatoid arthritis, ischemic diseases such as arteriosclerosis, and initial cancer growth. Here, we review features of NIF FI and photodynamic therapy for liver tumors.

## Near-Infrared Fluorescence Imaging During Liver Surgery Navigation

### Tumor Visualization

Gotoh et al. (13) and Ishizawa et al. (14) reported the novel technics for liver cancers with ICG fluorescence image (FI) navigation, which is based on the capacity of hepatocellular carcinoma (HCC) to accumulate ICG. ICG was injected several days before surgery. ICG FI found the HCC tumors that were completely removed, with negative margins, using the FI as a guide. It is essential to detect small HCCs for curative resection and improvement of patient outcomes. Further strong definition between tumor and non-tumor cells is essential for obtaining safe surgical margins. The authors showed that FI following pre-operative intravenous injection of ICG confirmed HCCs and colorectal liver metastases (CRLM) on cut surfaces of resected specimens in 37 patients with HCC and 12 with CRLM (14). There were three types of fluorescence patterns in tumors: (i) total, all tumor tissue showed uniform fluorescence; (ii) partial, some showed fluorescence; and (iii) rim, the surrounding liver parenchyma except cancer tissues showed fluorescence. Total-type tumors contained all well-differentiated HCCs, while rim-type tumors contained poorly differentiated HCCs and CRLM. In addition to detecting the small HCCs, ICG-FI was reportedly useful for the detection of CRLM (15, 16), peritoneal dissemination (17, 18), and lymph node metastasis from HCC (19).

The mechanism of ICG-FI for HCC has been revealed by gene expression and histochemical staining analyses (20). The expression of portal uptake transporters of ICG, which are organic anion-transporting polypeptide-8 and Na^+^/taurocholate cotransporting polypeptide (21), were well-maintained in differentiated HCC tissues. However, functional biliary excretion was disordered, which was followed by ICG retention in cancerous tissues at the time of surgery. In contrast, portal uptake transporters were downregulated in poorly differentiated HCCs and biliary excretion of ICG by surrounding non-cancerous hepatic parenchyma was disordered, resulting in rim-type fluorescence. In CRLM, the rim-type fluorescence signal is reportedly found by immature hepatocytes with reduced bile excretion (22).

**Figure 1:**
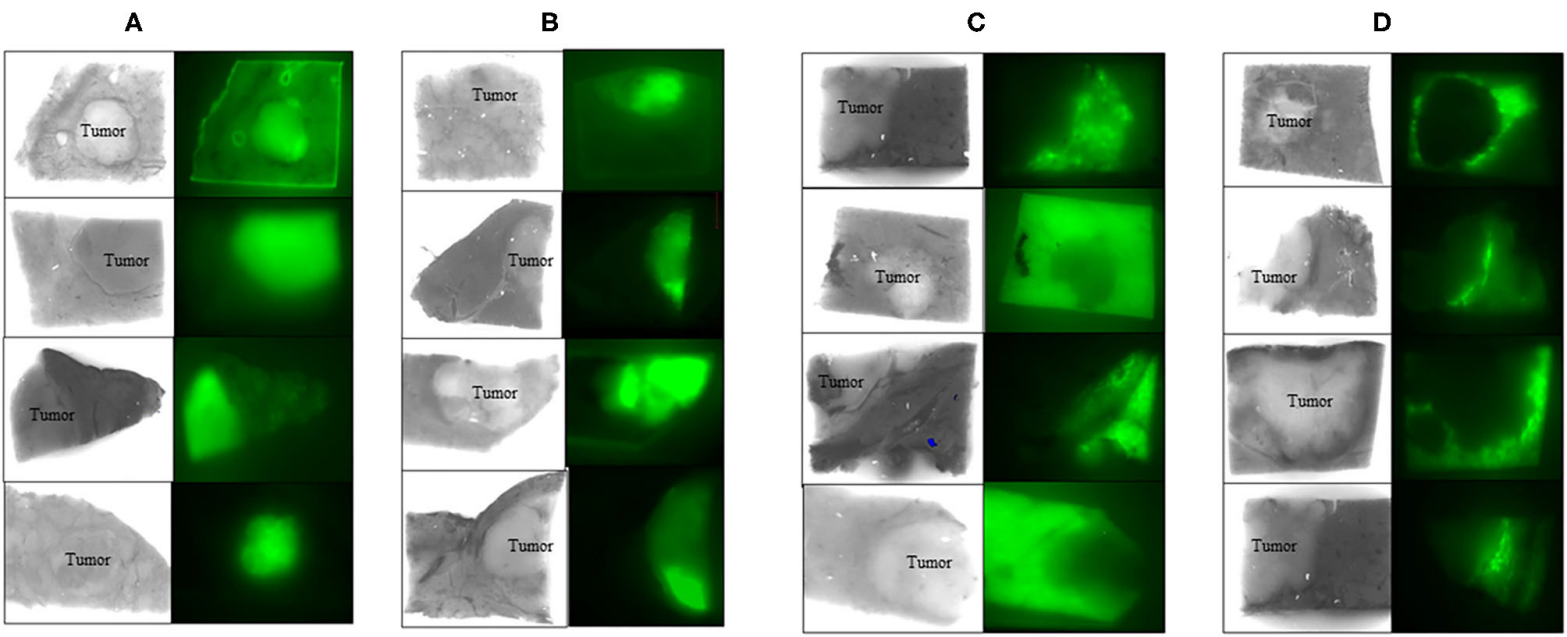
ICG fluorescence image in cancerous liver tissue. Whole **(A)** and partial **(B)** fluorescence image (FI) in cancer. Whole **(C)** and ring **(D)** FI in the surrounding non-cancerous liver tissue. ICG FI in resected liver specimens could confirm HCC. FI; filter excitation: 785 nm, emission: 845 nm, using Clairvivo OPT, Shimadzu, Kyoto, Japan.

We reported that ICG FI for HCC is effective for both assessing tumor differentiation and detecting fibrosis in non-cancerous liver parenchyma (23). We evaluated ICG FI of resected specimens from 190 HCC patients. This system (fluorescence image; using Clairvivo OPT, Shimadzu, Kyoto, Japan) consisted of a horizontal sample table, excitation light sources, charge-coupled device camera, camera lens, optical filter, and white laser-emitting diode light sources that illuminated the sample from four angles. They were classified into two groups: high cancerous [HC] and high surrounding [HS] groups, according to FI (Figure 1). These groups were further sub-classified into whole and partial types (Figures 1A,B), and whole and ring types (Figures 1C,D), respectively. The patients in the HC group had a higher prevalence of esophageal or gastric varices, and worse liver function than those in the HS group. The HC group also had a higher percentage of limited resection cases as compared with the HS group. Cirrhotic liver histology was more common in the HC group than in the HS group. In multivariate analysis, HC status was a predictive factor for cirrhosis in HCC patients. In the HC group, a higher number of well-differentiated HCC cases was seen in the partial-type subgroup [23/48 (48%), Figure 1A] than in the whole-type subgroup [7/68 (10%), Figure 1B]. In the HS group, a higher number of poorly differentiated HCC cases was seen in the ring-type subgroup [6/37 (16%), Figure 1C] than in the whole-type subgroup [0/37 (0%), Figure 1D]. Tumor differentiation and fibrosis in non-cancerous liver parenchyma could affect ICG FI in HCC. ICG FI may be a good indicator of fibrosis stage.

Intraoperative ICG-FI in hepatic tumors is useful for identifying subcapsular lesions for removal during laparoscopic hepatectomy, in which visual inspection and palpation are limited compared with open surgery (24). This technique has potential drawbacks, however, including a relatively high false-positive rate (14, 20). Lesions that are newly detected by ICG-FI should be resected only when other modalities, such as palpation and intraoperative US, identify them as tumors to be removed. The incidence of false positives can be reduced by not administering ICG on the day before surgery, especially in patients with decreased liver function due to cirrhosis or pre-operative chemotherapy (14). Detecting liver tumors through ICG FI involves visualizing cholestasis inside or around liver cancer because ICG FI does not truly image cancer cells. When new fluorescent regions are visualized during surgery, palpation and US for liver tumors should be performed. Additional resection should be considered if these extra methods suggest malignancies. As ICG FI is also limited in its observation depth (up to about 8 mm), intraoperative US is required to confirm findings that lie deeper in the liver.

**Figure 2:**
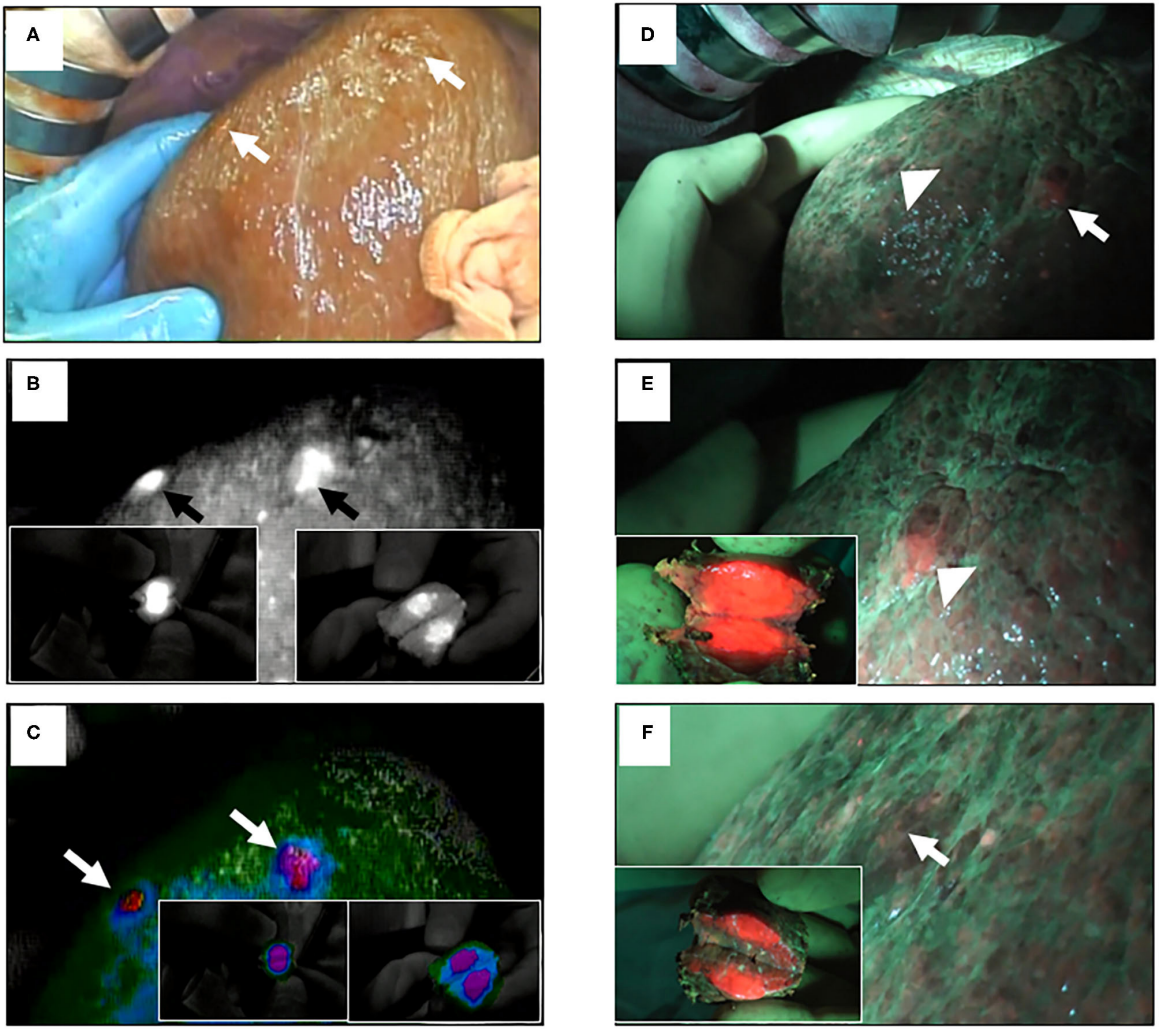
ICG and 5-ALA fluorescence images of superficial malignant liver tumors. **(A)** Two superficial malignant liver tumors with serosa (arrows); conventional white light. **(B)** ICG FI of the same tumors (arrows); insets show the incised lesions. **(C)** ICG FI of the same tumors using color mode (arrows). **(D)** The same tumors (arrowhead and arrow); blue light. **(E)** 5-ALA FI of the same tumors (arrowhead); inset shows the incised lesion. **(F)** 5-ALA FI of the other tumor (arrow); inset shows incised lesion.

**Figure 3:**
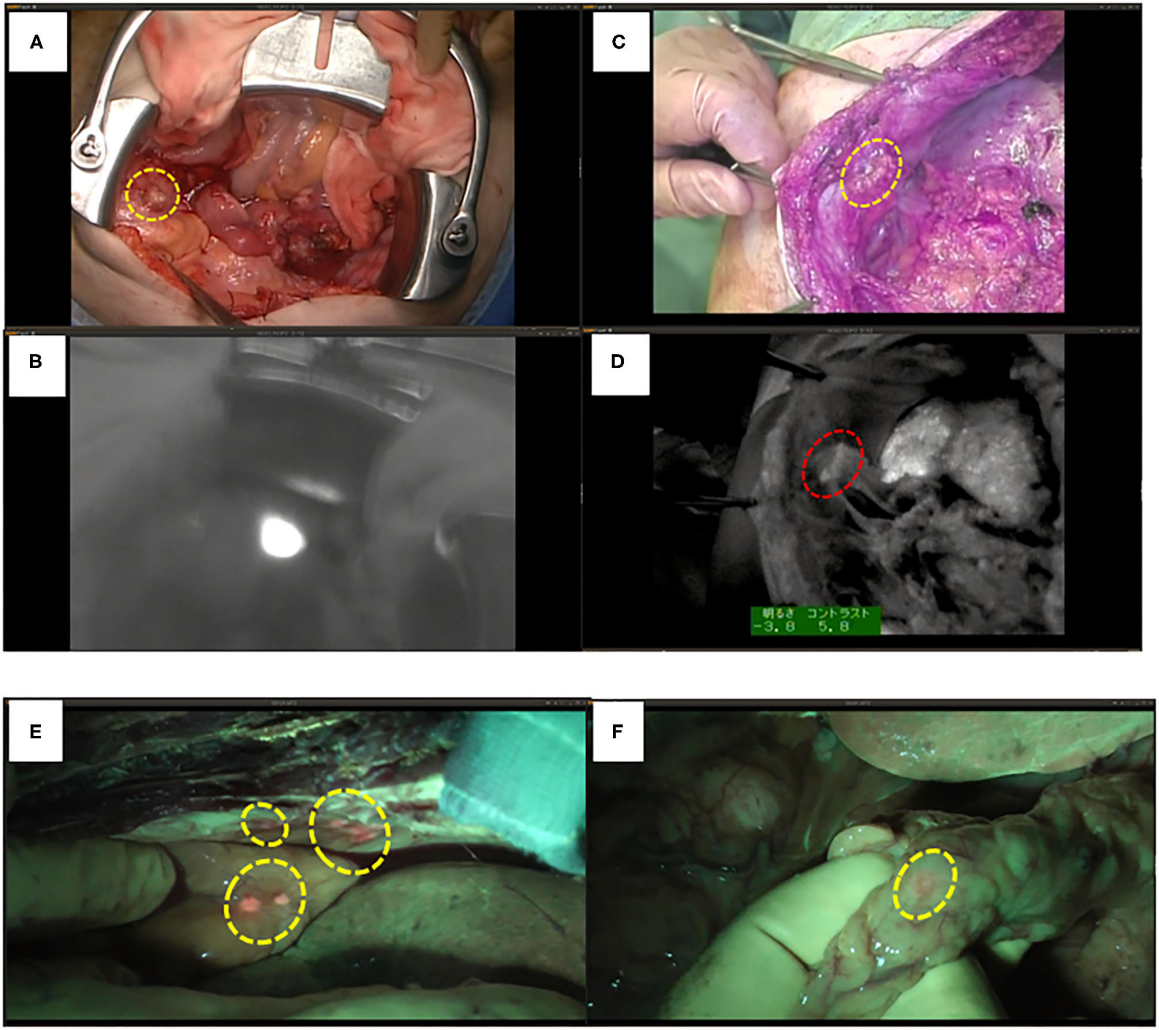
ICG and 5-ALA fluorescence images of metastases through HCC. **(A)** Ovarian and peritoneal metastases (yellow circle); conventional white light. **(B)** ICG FI of peritoneal metastasis. **(C)** Peritoneal metastasis (yellow circle); conventional white light. **(D)** ICG FI of peritoneal metastasis (red circle). **(E)** 5-ALA FI of peritoneal and colon metastases of intrahepatic cholangiocellular carcinoma (yellow circles). **(F)** 5-ALA FI of omental metastasis (yellow circle).

By using ICG and 5-ALA FI, the latent, superficial, and small liver tumors were detected and treated pre-operatively and/or intraoperatively, as we reported previously (25). 5-ALA is a natural precursor of heme, and is accumulated and converted to protoporphyrin IX, which emits red fluorescence in cancer cells (26). Thus, 5-ALA is used as a photosensitizer in photodynamic diagnostics for neurosurgery and urology (27–30). Imaging with ICG or 5-ALA detected small latent liver tumors (Figure 2) and peritoneal dissemination (Figure 3). We conducted a study with 48 patients who had primary liver tumors within 5 mm of the liver surface. With ICG, the sensitivity, specificity, and accuracy for detecting the pre-operatively identified primary tumors were 96, 50, and 94%, respectively. Twelve latent, small tumors were newly detected on the liver surface using ICG. Five of these tumors were determined to be carcinomas. With 5-ALA, the sensitivity, specificity, and accuracy for detecting the primary tumors were 57, 100, and 58%, respectively. Five latent, small tumors were newly detected using 5-ALA; all were carcinomas. Overall, five new tumors were detected by both ICG and 5-ALA FI: two were HCCs and three were metastases of colorectal cancer. The sensitivity and specificity of ICG FI for primary tumor detection were relatively high and low, respectively, but the opposite was true for 5-ALA imaging. In another study, Inoue et al. reported that there was 100% sensitivity for detecting HCC, 86% for colorectal liver metastases, and 100% for liver tumors of various etiologies. 5-ALA showed an overall sensitivity of 93% for detection in their experience with a series of 70 patients who underwent hepatic resection (31). However, Boogerd et al. reported that their study was designed to compare FI and conventional imaging modalities for laparoscopic detection of both primary and metastatic tumors in the liver (32). Sensitivity for various modalities was 80% (CT), 84% (MRI), 62% (inspection), 86% (laparoscopic ultrasonography), and 92% (ICG-FI). All 26 malignancies that were examined could be detected by combining laparoscopic ultrasonography and ICG-FI (100% sensitivity). To the best of our knowledge, our study is the first to detect liver tumors using both ICG and 5-ALA FI. FI using both ICG and 5-ALA may provide greater specificity in detecting surface-invisible liver tumors than using ICG FI alone. Both imaging methods may help improve treatment strategies for patients with liver tumors.

### Cholangiography

Cholangiography is used to visualize the biliary tract intraoperatively to avoid damage to the bile ducts (33). Routine cholangiography is recommended for cholecystectomy (34, 35). A multicenter randomized controlled trial confirmed the superiority of extrahepatic bile-duct identification using ICG fluorescent cholangiography in laparoscopic cholecystectomy (36). Because ICG binds to human bile proteins, such as albumin and lipoproteins (8), intra-bile-duct injection of ICG enables FI of the biliary tract (37). The fluorescent intensity of ICG bound to these proteins was ~0.25 mg/mL and correlates with its concentration. However, it was found to decrease at high concentrations from the absorption of near-infrared light by the ICG (5). Therefore, a diluted ICG solution (~0.025 mg/mL) should be injected into the bile duct to obtain a clear fluorescent image of the bile duct (31). ICG was detected in the bile duct 20 min after injection and maintained for up to 2 h in patients who underwent laparoscopic cholecystectomy (38, 39). In donor hepatectomy and laparoscopic hepatectomy, ICG cholangiography has been reported (40, 41). Recently, Hong et al. reported that ICG fluorescent cholangiography was successful in delineating the biliary system around the hilar plate and in determining optimal bile duct division points during laparoscopic donor hepatectomy in their ten patients. They suggest that this technique may be particularly useful in laparoscopic surgery, during which intraoperative cholangiography and probing through the divided bile duct opening or cystic duct opening is relatively difficult (42). ICG fluorescent cholangiography is also useful for real-time detection of bile leaks during hepatectomy (43, 44).

**Figure 4:**
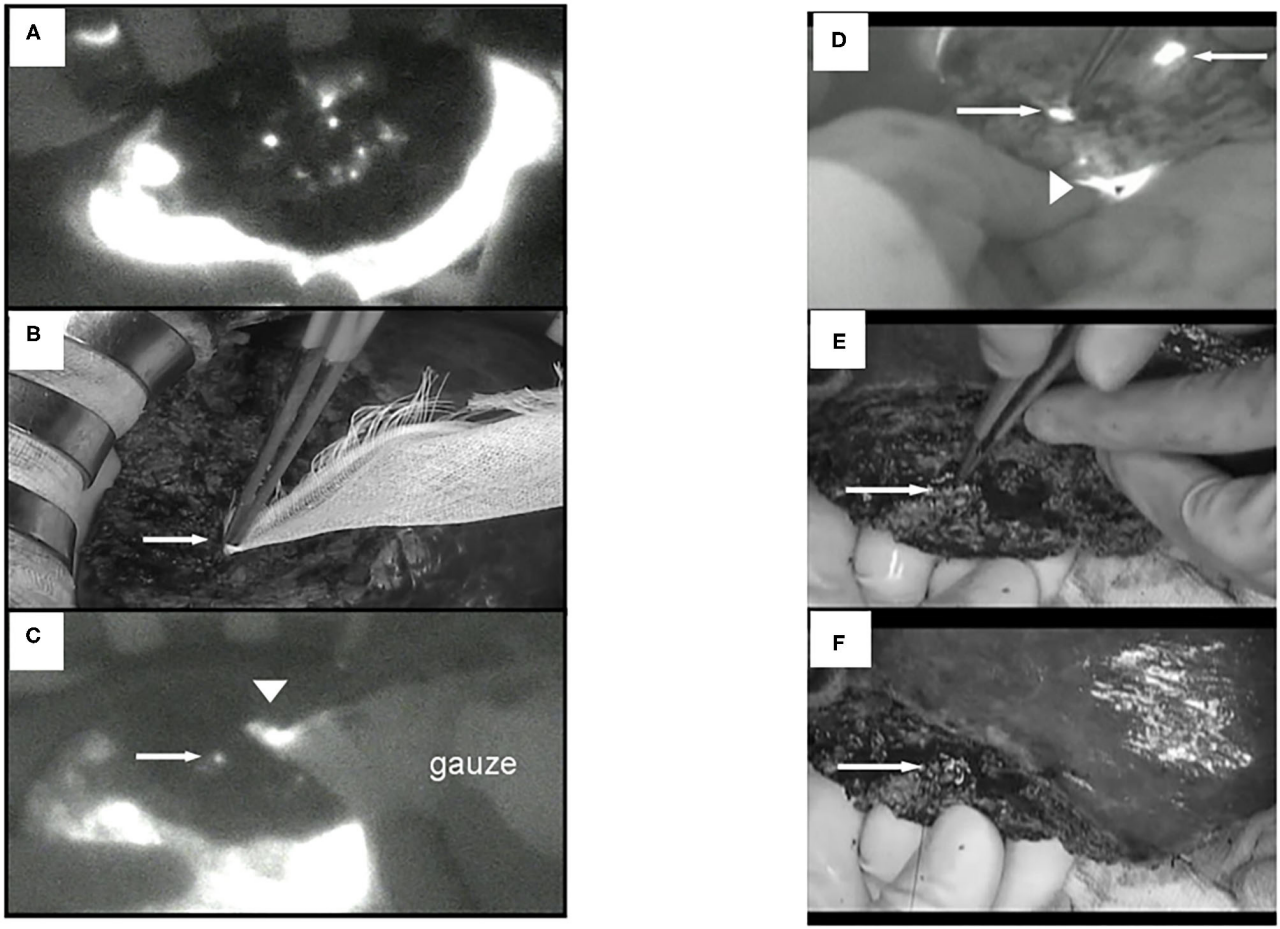
ICG fluorescent images by the photodynamic eye system. **(A)** White spots (possible bile leakages) on cut liver surface. **(B)** Minor leak from duct is compressed with gauze, which is not visible to the surgeons (arrow). **(C)** ICG FI after gauze application reveals minor leakage on the cut liver surface (arrow); fluorescence is also detected through the gauze (arrowhead). **(D)** FI identified two fluorescing ducts on the cut liver surface (arrows). The upper bile duct is intact (upper arrow). Arrowhead: common bile duct. **(E)** The lower fluorescing lesion corresponds to a partly closed bile duct stump (arrow). **(F)** The bile duct stump was repaired (arrow, z-sutures using 6-0 non-absorbable thread).

We have performed a randomized controlled trial with ICG fluorescence cholangiography in 102 patients who underwent hepatic resection without biliary reconstruction. The patients were divided into two groups: with or without ICG fluorescence cholangiography. In the control group, five patients developed post-operative bile leakage, but in the ICG cholangiography group, no bile leakage was observed in any patient (10 vs. 0%, *P* = 0.019; Figure 4) (44). Thus, ICG cholangiography may help prevent bile leakage. ICG cholangiography also prevented bile leakage and bile duct stenosis in bile duct anastomosis in living-donor liver transplantation (42, 45).

### Imaging of Hepatic Segments

Fusion ICG FI, which displays both fluorescence images and macroscopic views on a single screen, provides clear demarcation lines for more accurate parenchymal dissection (46). Nishino et al. showed that the projection mapping with ICG fluorescence detected anatomical landmarks for parenchymal dissection in clinical utility (47). Makuuchi et al. first reported anatomical liver resection (48), injecting indigo-carmine into the portal vein, which was followed by some studies of anatomical liver resection using non-anatomical liver resection (49–52). Aoki et al. obtained fluorescence images of the liver surfaces using an infrared camera, after portal vein branches in the HCC-bearing hepatic segment were punctured by intraoperative US (53, 54). They reported that the ICG-staining technique showed the sub-segments and segments in 90% (73/81) of patients, and that there were no differences in detection rates for liver segment boundaries between non-cirrhotic and cirrhotic livers.

Laparoscopic liver resection for HCC was established and became a standard treatment for minor liver resection (55). It is also performed in specialized centers for laparoscopic liver surgery (56). Ishizawa et al. used the ICG staining technique for laparoscopic anatomical resection (57). Ueno et al. used laparoscopic liver segmentation by interventional radiology technique (58). Although the ICG FI method is a relatively simple method, in the positive staining method when there are multiple dominant portal vein branches or when it is difficult to puncture a thin vessel of 2 mm or less, it is not possible to accurately visualize the hepatic segments. In the negative staining method, prior treatment of the dominant portal vein branch is required, but it is necessary to carefully perform the peeling operation while paying sufficient attention to vascular and bile duct injury (59). Additionally, unlike indigo-carmine which disappears in a short time, the fluorescent region from ICG is maintained for a long time, so it is difficult to correct the stained region when it is incorrect. To obtain the desired accurate ICG FI during the operation, it is important to perform sufficient simulation before surgery and carefully plan the appropriate ICG FI method for each case.

ICG FI can apparently be safe and reliable for hepatectomy at many facilities and is expected to help improve liver cancer treatment, though its usefulness should be verified in a large-scale, multicenter study.

## Photodynamic Therapy for Liver Tumors

### Indocyanine Green-Lactosome Has Anti-neoplastic Effects for HCC

We have developed a macromolecular micelle (ICG-lactosome, ICG-L) formed by self-assembly of a novel amphipathic polydepsipeptide (containing hydrophilic and hydrophobic parts). ICG-L does not accumulate very much in internal organs because of the stealth effect, but it selectively accumulates in cancer tissues with higher concentration because of the enhanced permeability and retention effect (60). In polydepsipeptide, polysarcosine constitutes the hydrophilic portion, comprises amino acids found in living tissue, and has hydrophilicity equal to or greater than that of polyethylene glycol. It is biodegradable and is widely used in industrial and medical materials. In contrast, polylactic acid is the hydrophobic portion, also biodegradable and used as a medical material, frequently in bone resorbable applications. Compared with other conventional biocompatible nanoparticles, ICG-L is easy to use in designed products. Its particle sizes can be adjusted and modified with biomaterials such as peptides; it may work as a carrier for “theranostic” diagnostic agents and pharmaceuticals (61).

**Figure 5:**
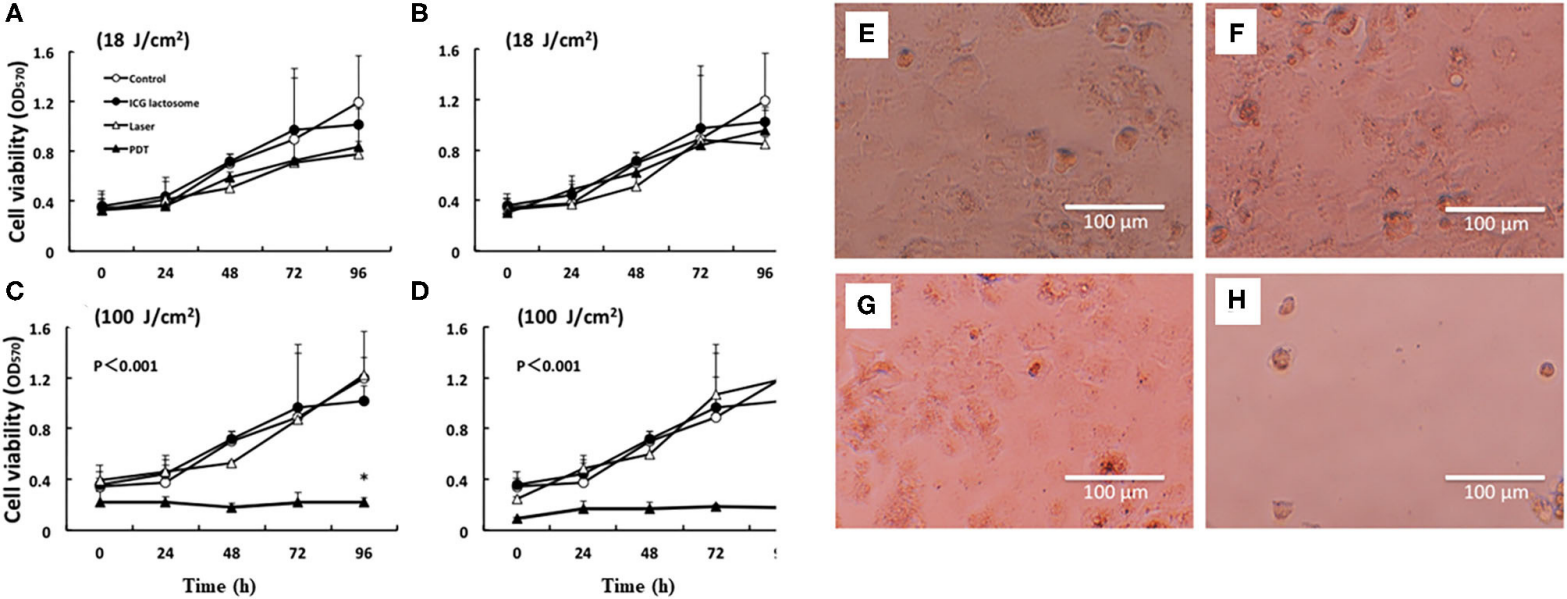
*In vitro* PDT on cell viability and morphological changes in ICG lactosome-treated HuH-7 cells. Cells were divided into four groups: control, ICG-L, laser, and PDT (ICG-L + laser). The laser and PDT groups were irradiated with **(A)** 18 J/cm^2^ (190 mW/cm^2^ and 95 s), **(B)** 18 J/cm^2^ (340 mW/cm^2^ and 55 s), **(C)** 100 J/cm^2^ (190 mW/cm^2^ and 525 s), and **(D)** 100 J/cm^2^ (340 mW/cm^2^ and 300 s). Cell viability (OD570) was measured by the MTT assay (*n* = 4 /time/group; *P < 0.001 for ICG-L vs. other groups). Morphological changes (phase-contrast microscope at 96 h). **(E)** Control, **(F)** ICG-L, **(G)** laser [100 J/cm^2^ (340 mW/cm^2^ and 300 s)], and **(H)** PDT (ICG-L + laser). https://doi.org/10.1371/journal.pone.0183527.g001.g002.

For the evaluation of PDT efficacy *in vitro*, HuH-7 (human HCC cell line) cells were treated with ICG-L or ICG, followed by PDT. The cell viabilities were then measured. Laser irradiation revealed that HuH-7 cells with ICG-L showed toxic effects, but the cells with ICG alone did not (Figure 5).

**Figure 6:**
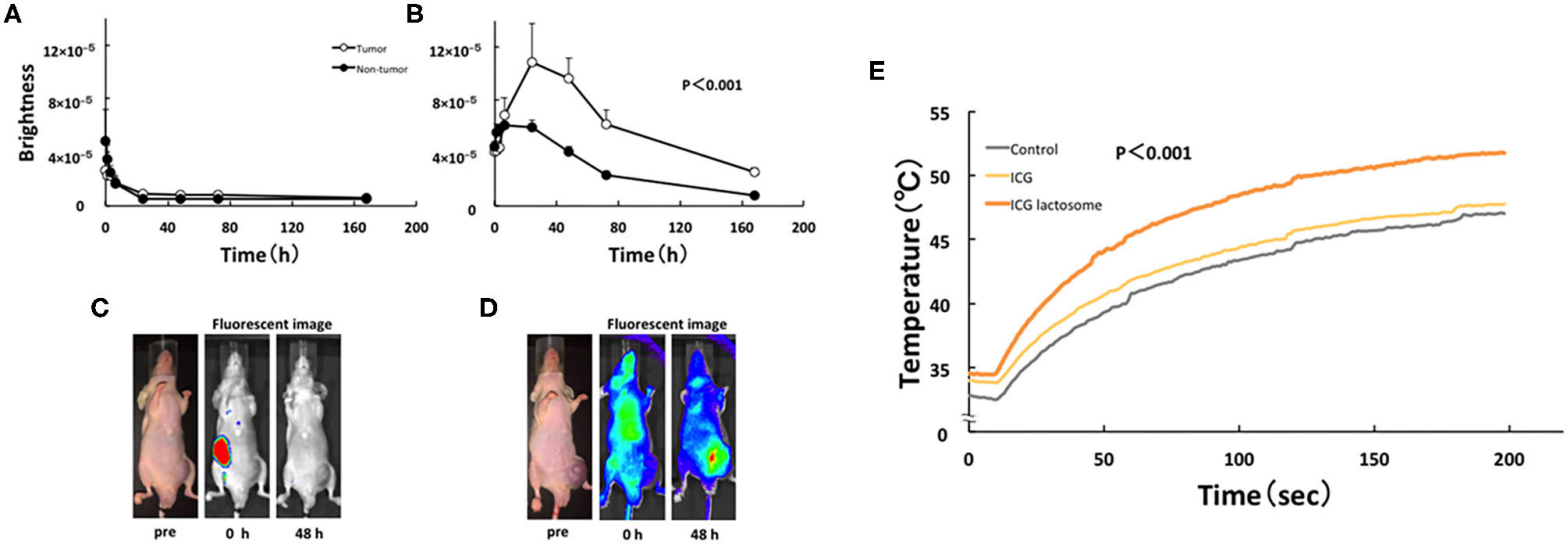
*In vivo* fluorescence imaging in ICG lactosome-treated mice with subcutaneous tumors. After injection of ICG **(A,C)** or ICG-L **(B,D)** in mice, the brightness of the tumor (open circles) and non-tumor (contralateral inguinal, closed circles) areas was measured (IVIS system). *P* < 0.001 between the tumor and non-tumor regions in the ICG-L group; *n* = 5/group. **(E)** Effect of laser irradiation on tumor temperature. At 48 h after ICG (yellow), ICG-L (brown) or no injection (control, dark) in the tumor implanted-mice, laser irradiation (500 mW/cm^2^ and 200 s, 100 J/cm^2^) was initiated, and the temperatures were measured (*P* < 0.001 for ICG vs. ICG-L; *n* = 6/group). https://doi.org/10.1371/journal.pone.0183527.g004,g006.

**Figure 7:**
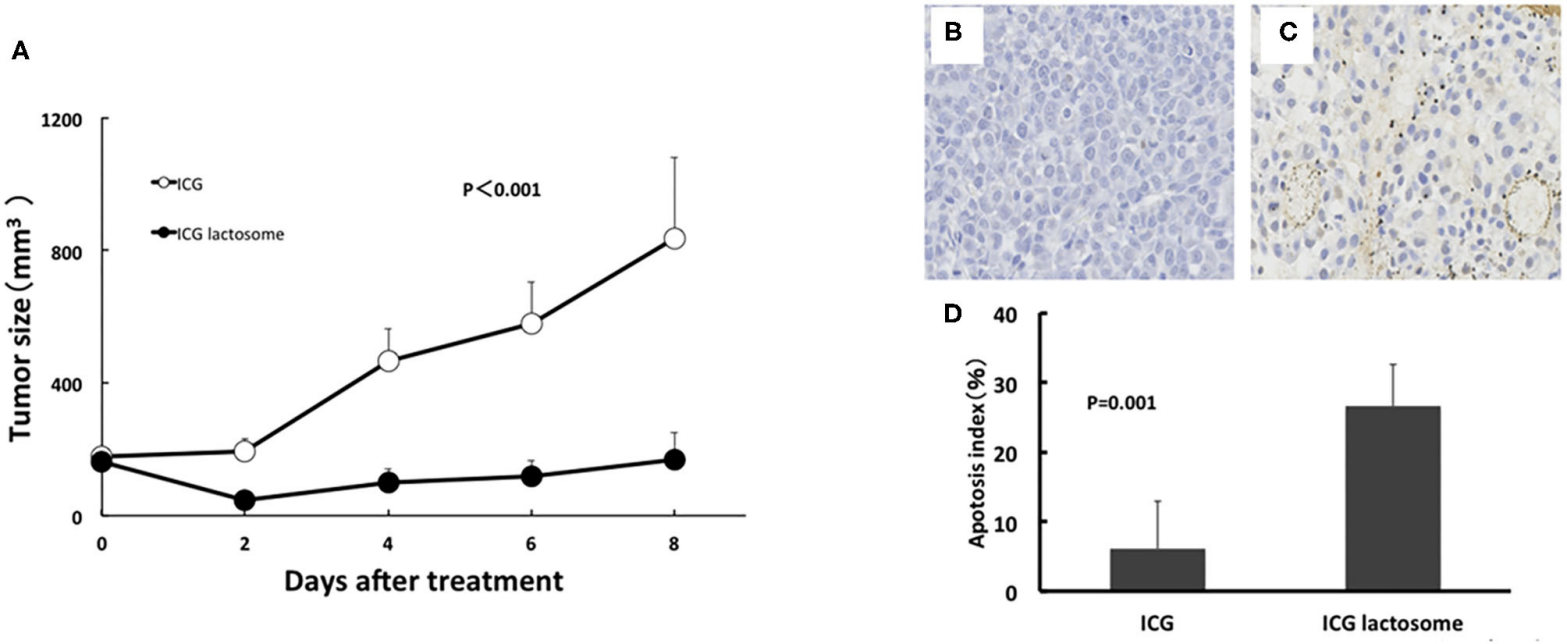
*In vivo* PDT on tumor growth in ICG lactosome-treated mice with subcutaneous tumors. **(A)** At 48 h after ICG (open circles) or ICG-L (closed circles) injection in mice, laser irradiation (500 mW/cm^2^ and 200 s, 100 J/cm^2^) was carried out, and tumor volumes were measured. (*P* < 0.001 for ICG vs. ICG-L; *n* = 6/group.) **(B–D)** Effect of PDT on apoptosis of tumors. Tumors were resected at 24 h after irradiation (500 mW/cm^2^ and 200 s, 100 J/cm^2^) in the ICG **(B)** and ICG-L **(C)** groups and examined for apoptosis (TUNEL staining). **(D)** Apoptotic indexes in the ICG-treated and ICG L-treated groups (*P* = 0.001 for ICG vs. ICG-L; *n* = 5/group.) https://doi.org/10.1371/journal.pone.0183527.g005,g007.

For NIF *in vivo*, BALB/c nude mice were subcutaneously treated with HuH-7 cells and then intravenously with ICG-L or ICG. These transplanted animals were treated with PDT and the antineoplastic effects were analyzed. Indeed, NIF imaging revealed that the tumor-area fluorescence in ICG-L-treated animals was higher than that of contralateral regions at 24 h after injection and thereafter (Figures 6B,D), although there was no difference between those in the tumor-area fluorescence in ICG-treated animals (Figures 6A,C). PDT had continuous phototoxic effects in the ICG-L group (Figure 7A). By laser irradiation, the median temperature in the tumor areas increased from 34 to 47.7°C and from 34.5 to 51.7°C at 0–200 s in the ICG-L and ICG groups, respectively (Figure 6E), where the median temperature in the ICG-L group was higher than that in the ICG group.

In the former, there was substantial apoptosis with a higher apoptotic index in the ICG-L group than in the ICG group (Figures 7B–D). The mechanisms involved in tumor suppression by PDT in the ICG-L group were presumably caused by singlet oxygen and ICG degradation products generated by a photochemical reaction (62). Singlet oxygen can be generated as ICG is exposed to near-infrared excitation light. The decomposition products that can be produced by ICG can further decrease cell viability (63). These products may lead to tumor cell apoptosis. PDT with ICG-L increased the tumor suppression by the heat generated from a photothermal reaction. During *in vivo* analysis of PDT with ICG-L, the temperature increased (50°C and above) relative to ICG alone, suggesting that the heat may be sufficient to damage tumor growth. In addition, optimization of ICG-L dosage and laser irradiation fluence are also necessary to prevent future tumor growth. The temperature increase is a major factor in the antitumor effect obtained in our study because PDT using ICG reportedly has a thermal effect (64). The temperature increase by PDT likely depends on various photosensitizers, including ICG. PDT using ICG can facilitate a temperature increase with antitumor effects, which supports the usefulness of ICG-L.

Our results demonstrated that tumor cells transplanted in BALB/c nude mice incorporated ICG-L and that PDT had antineoplastic effects. NIF imaging and PDT with ICG-L may be useful diagnostic and/or therapeutic strategies for HCC.

There are some limitations to ICG FI. First, ICG-L may possibly accumulate in both malignant and benign tumors, causing excessive influence in undesired areas by PDT. There is little knowledge regarding angiogenesis in benign tumors compared with in malignant tumors. Because benign tumors generally have slower growth rates and do not infiltrate into other tissues compared with malignant tumors, we believe it would be difficult to integrate ICG-L into benign tumors.

In clinical situations, Ishizawa et al. reported that ICG FI can be used to observe HCC lesions based on their tumorous fluorescence. This is because the portal uptake of ICG is preserved in well- or moderately differentiated cancer tissues, despite the lack of biliary extraction and to delineate poorly differentiated HCC as rim-fluorescing lesions because of biliary excretion disorders in the surrounding non-cancerous liver tissues that are compressed by the tumor (14).

The second limitation is that it is not clear how the PDT effect of ICG-L depends on the tumor differentiation, tumor size, and depth from the liver surface. We have only investigated the Huh-7 cell line as well-differentiated HCC. ICG-L (excitation wavelength: 774 nm, fluorescence wavelength: 805 nm) reaches a depth of 10–20 mm subcutaneously in the near infrared region (700–100 nm) (60, 61). Future studies are needed on the PDT effect of ICG-L on the tumor diameter and degree of HCC differentiation.

Funayama et al. (65–67) reported that PDF with ICG-L delayed the development of paralysis in rats with spinal metastases. Tsujimoto et al. (68, 69) reported that PDF with ICG-L inhibited the growth of metastases. Recently, we found an antitumor effect of PT with ICG-L on gallbladder cancer in xenograft tumors (70). In other experiments, Kaneko et al. (71, 72) reported using PDT with ICG and an NIR laser for HCC. HuH-7 cells were transplanted subcutaneously into BALB/c-*nu/nu* mice and ICG was administered 24 h before NIR irradiation (days 1 and 7). Repeated NIR increased the cell toxicity of ICG-NIR therapy. They also found that ICG-NIR therapy increased apoptosis in tumor cells through a photothermal effect and oxidative stress.

## Conclusions

ICG FI has effects on fibrosis detection and tumor differentiation measurement in non-cancerous liver parenchyma in HCC. ICG FI can be a good indicator of fibrosis stage and apparently detect smaller liver tumors. FI with 5-ALA provided higher specificity in detecting surface-invisible liver tumors as compared with FI with ICG. Usage of both ICG and 5-ALA imaging can potentially improve treatment strategies for patients with liver tumors. Simply put, FI offers the benefits of a higher-resolution intraoperative navigation tool for liver cancer. ICG-L is useful as a diagnostic and therapeutic agent for HCC.

PDT using ICG-L, which is repeatedly performed, can be used in endoscopic or laparoscopic therapy for small HCC tumors. To develop PDT with FI in clinical applications, we will examine optimal drug dosages, irradiation conditions, and the effects of treatment on other carcinomas.

## Author Contributions

MK drafted and wrote the manuscript. HM, MI, HK, KM, TT, HH, TO, and MS participated in the study design and helped draft the manuscript. All authors contributed to the interpretation of the findings, read, and approved the final manuscript.

## Conflict of Interest

The authors declare that the research was conducted in the absence of any commercial or financial relationships that could be construed as a potential conflict of interest.

## Abbreviations

5-ALA: 5-aminolevulinic acid
CLRM: colorectal liver metastasis
CT: computed tomography
FI: fluorescence imaging
HC: high cancerous (fluorescence in cancerous tissue)
HS: high surrounding (fluorescence in surrounding tissue)
ICG: indocyanine green
ICG-L: ICG-lactosome
ICGR15: ICG retention rate at 15 min
MRI: magnetic resonance imaging
NIR: near-infrared
PDT: photodynamic therapy
US: ultrasonography.
